# Prevalence, risk factors and mixed Eimeria infection detection in broiler and Sonali birds of some poultry concentrated zones of Bangladesh

**DOI:** 10.1007/s00436-026-08728-9

**Published:** 2026-07-16

**Authors:** Bimal Chandra Karmakar, Nusrat Nowrin Shohana, Subir Sarker, Anita Rani Dey, Sharmin Aqter Rony, Shirin Akter, Anisuzzaman, Mohammad Zahangir Alam

**Affiliations:** 1 Department of Parasitology, Faculty of Veterinary Science, Bangladesh Agricultural University, Mymensingh, 2202 Bangladesh; 2 Bangladesh Academy for Rural Development (BARD), Cumilla, Bangladesh; 3 Biomedical Sciences and Molecular Biology, College of Medicine and Dentistry, James Cook University, Townsville, QLD 4814 Australia

**Keywords:** Chicken coccidiosis, Prevalence, Risk factors, Mixed infection

## Abstract

Chicken coccidiosis is a ubiquitous and economically significant disease that causes substantial production losses in the poultry industry. It is caused by protozoan parasites of the genus *Eimeria*, which damage the intestinal epithelium, leading to diarrhoea, poor growth, and mortality. This study investigated the prevalence, risk factors, molecular diversity, and phylogenetic relationships of *Eimeria* infections in commercial poultry farms in Bangladesh. A total of 350 fresh fecal samples were collected from seven major poultry-producing regions and screened microscopically for oocysts. Farm-level data were obtained through structured interviews to assess associated risk factors. Samples with high oocyst loads were subjected to molecular analysis targeting the internal transcribed spacer 1 region, followed by Sanger sequencing and phylogenetic reconstruction. The overall prevalence was 85.14%, with regional variation ranging from 61.22% in Sylhet to 100% in Joypurhat. Significant associations (*p* < 0.05) were observed with bird type, prior coccidiosis exposure, drinking water source, anticoccidial used, and veterinary support. Sequence analysis confirmed the presence of all seven recognized *Eimeria* species, showing 98.05–100% similarity with reference sequences. Mixed infections were highly prevalent (97.57%), with multiple species detected per sample. Phylogenetic reconstruction showed that the study isolates consistently grouped with their corresponding reference taxa, supported by strong bootstrap values, and confirming accurate species identification and genetic relatedness. In conclusion, detection of all *Eimeria* species, together with the high burden of mixed infections and the influence of modifiable management factors, underscores the necessity for improved and integrated control measures for coccidiosis in commercial poultry systems in Bangladesh. Clinical trial number: not applicable.

**Supplementary Information:**

The online version contains supplementary material available at https://doi.org/10.1007/s00436-026-08728-9.

## Introduction

Chicken coccidiosis, caused by the obligate intracellular protozoa under the genus *Eimeria*, remains one of the most significant threats to the global poultry industry (Lee et al. 2022). The disease is responsible for annual losses exceeding US$14 billion due to costs associated with prevention, treatment, and reduced productivity (Blake et al. 2020). There are seven common species of Eimeria, like E. acervulina, E. brunetti, E. mitis, E. maxima, E. necatrix, E. praecox, and E. tenella, which cause chicken coccidiosis (Liao et al. 2024). Each species of Eimeria parasite has a specific predilection site and pathogenicity in the chicken intestine, resulting in varying degrees of clinical manifestations (Attree et al. 2021). Eimeria parasites disrupt gut homeostasis by damaging enteric cells and causing malabsorption and they also catalyze various forms of subclinical and clinical coccidiosis, which are responsible for increased mortality and morbidity, and predispose to other enteric pathogens (Chapman 2014; Yang et al. 2020).

Broiler chicken is popularly reared for meat purpose all over the world. Sonali chicken, for its phenotypic similarities to local birds, better productive performance and well adapted nature, has become most popular among the people of Bangladesh (Jahan et al. 2021). It is developed by cross breeding the male of Rhode Island Red (RIR) and female of Fayumi chicken. Initially it was introduced in the northern part of Bangladesh (Prank et al. 2024) through a project funded by Danish Government namely Smallholder Livestock Development Project (SLDP-1 and SLDP-2). Currently, like broiler, Sonali poultry is reared throughout Bangladesh. However, Sonali Chickens are frequently infected with various infectious diseases among which coccidiosis is the most prevalent (Tipu et al. 2021; Prank et al. 2024; Aryal et al. 2025).

The global prevalence of coccidiosis varies widely, ranging from 7 to 98%, influenced by environmental conditions, management practices, and diagnostic approaches (Flores et al. 2022). In Bangladesh, reported prevalence rates are highly inconsistent, with values ranging from 13.78% to 82.29% across different districts (Iqbal et al. 2017; Rony et al. 2021; Datta et al. 2025; Islam et al. 2025). This variability highlights the need for region-specific epidemiological investigations. Besides, several farm-level factors, including bird age, production type, flock size, and litter management, have been identified as important determinants of infection (Prakashbabu et al. 2017; Liao et al. 2024). In essence, effective prevention and control of coccidiosis require a comprehensive strategy involving vaccination, incorporation of natural feed additives, prophylactic use of anticoccidial drugs, and the maintenance of optimal farm management practices (Broom 2021). Moreover, systematic studies integrating prevalence data with risk factor analysis are also crucial but remain inadequately explored in the Bangladeshi context.

Accurate identification of *Eimeria* species is essential for effective control strategies, as species differ in pathogenicity, immunogenicity, and anticoccidial sensitivity. Traditional microscopic identification based on oocyst morphology is widely used but suffers from limited specificity due to overlapping morphological characteristics, particularly in mixed infections, and requires substantial technical expertise (Jenkins et al. 2008; Haug et al. 2008; Kawahara et al. 2010). Previous studies in Bangladesh have reported multiple *Eimeria* species using morphological methods; however, inconsistencies between approaches and limited molecular characterization restrict a comprehensive understanding of circulating strains (Alam et al. 2020, 2021; Islam et al. 2025).

Molecular techniques, particularly PCR-based assays, offer enhanced sensitivity and specificity for species-level identification and detection of mixed infections, even at low parasite loads (Haug et al. 2008; Fatoba and Adeleke 2018, Alam et al. 2021). Especially, the internal transcribed spacer 1 (ITS1) region has been widely employed as a reliable genetic marker for differentiation and phylogenetic analysis of *Eimeria* species (Oliveira et al. 2011). Despite these advances, there is a notable lack of studies in Bangladesh that combine molecular identification, phylogenetic analysis, and epidemiological risk factor assessment within a single framework.

This study addresses this critical gap by integrating molecular and epidemiological approaches to provide a comprehensive characterization of *Eimeria* infections in commercial poultry farms in Bangladesh. Specifically, this study determines the prevalence and farm-level risk factors of *Eimeria* infection. Besides it detects different *Eimeria* species by ITS1-based PCR. Furthermore, the study also explores the phylogenetic relationships and mixed infections of *Eimeria* in the study farms.

## Materials and methods

### Fecal sample collection and oocyst identification

**Fig. 1:**
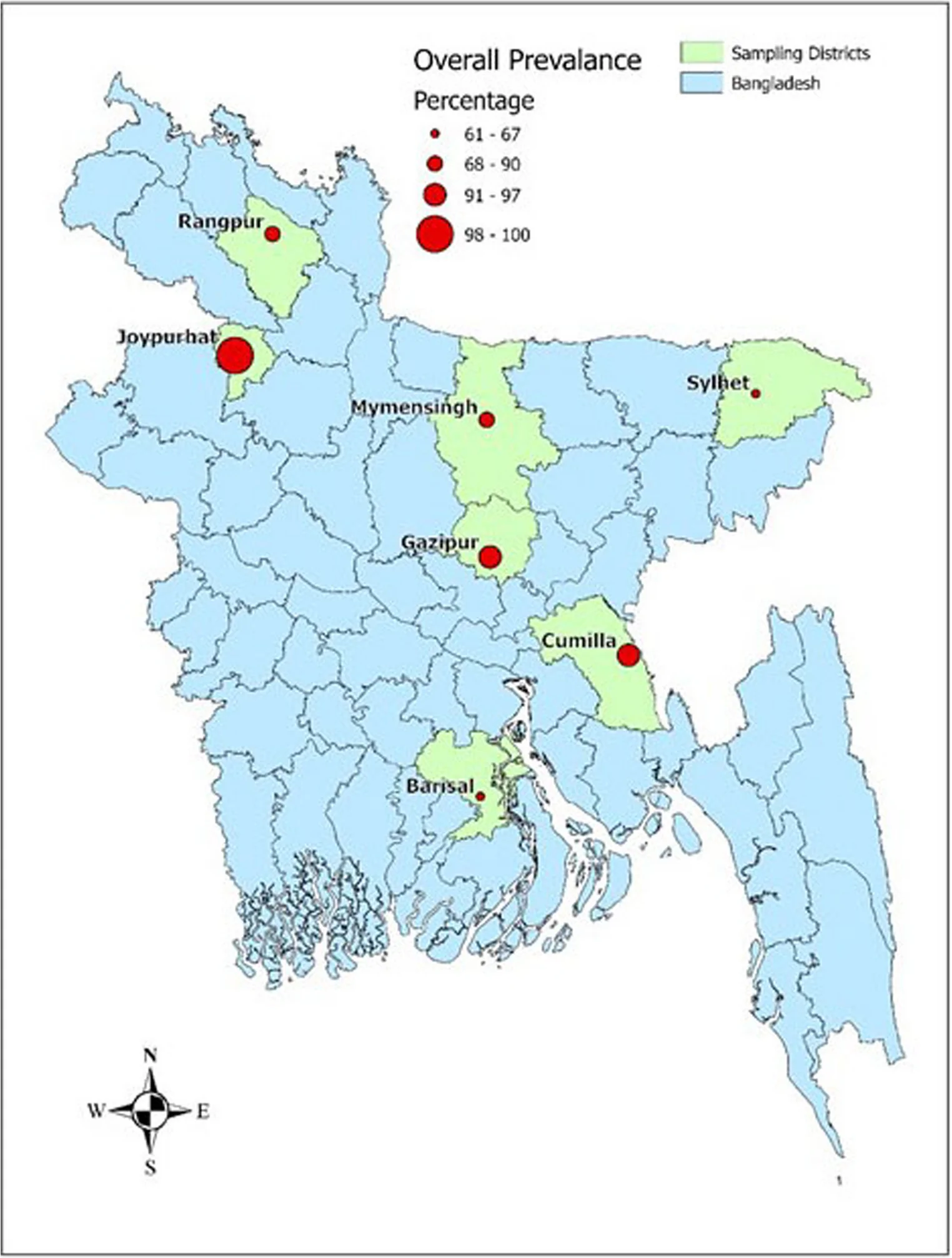
Geographic distribution of the study districts in Bangladesh. The highlighted (sky-colored) areas indicate the districts included in the study. The size of each circle represents the prevalence of *Eimeria* infection in the corresponding district

A total of 350 fresh fecal samples were collected from commercial poultry farms of seven poultry concentrated zones of Bangladesh, namely Mymensingh, Gazipur, Cumilla, Sylhet, Barisal, Rangpur, and Joypurhat (Fig. 1). The sample size was calculated by the equation given by (Thrusfield 1995). According to this formula sample size (n)= Z^2^[P(1-P)]/d^2^ Here, n=sample size, Z = The standard normal deviate for the desired confidence level. For a standard 95% confidence interval, (Z = 1.96). P=expected prevalence: 65% (*P* = 0.65), d= absolute precision: 5%=0.05. So, *n* = 349.44 **≈** 350. Given the low probability of *Eimeria* infection in birds below 15 days of age (Adhikari et al. 2008; Iqbal and Begum 2010), Sonali and Broiler farms with 15–60 days-old birds, reared on floor rearing system were purposively selected for fecal sample collection. However, for resh fecal samples collection farms with the aforementioned criteria were randomly selected. Following the methods described by Kumar et al. (2014), approximately 100 gm fresh fecal sample was collected from the ‘W’ shaped sites of the sheds to avoid sampling bias. Immediately after collection, each sample was placed in a pre-labeled polythene zipper bag and subsequently accumulated in a large, locally made insulated cork sheet box (thermos cool box) packed with ice and transferred to the laboratory. The fecal samples were processed by dilution with water and examined under a microscope to identify oocysts.

### Questionnaire survey

All the respective farmers were interviewed via face-to-face interview using this pretested structured questionnaire (Supplementary file 1) and thereby epidemiological data of farm management practices (types of birds reared, age of birds, litter used, litter disposal, source of feed and water, use of disinfectants, previous history of coccidiosis, farm size, floor type, biosecurity status, consultation services, etc.) were collected.

### Oocyst isolation and DNA extraction

Among the examined samples, 206 samples were further processed for molecular study. A slightly modified form of McMaster technique (Soulsby 1968) was used to determine the number of oocysts per gram of feces. If one gram of feces contains ≥ 50 oocysts of any species of *Eimeria*, it means at least 1 oocyst of any species of *Eimeria* in McMaster chamber, which is equivalent to ≥ 50 OPG. Then the poultry farm was considered positive for molecular analysis, otherwise, negative (Alemayehu et al. 2013). In our cases, when 10 oocysts in McMaster chamber (≥ 500 OPG) were considered for molecular analysis. Oocysts were isolated through floatation and centrifugation techniques described by Qi et al. (2020). Isolated oocyst samples were preserved in 2.5% potassium dichromate solution at room temperature for 5–7 days with sufficient aeration by stirring and stored at 4 °C until further processing (Ojimelukwe et al. 2018). To perform DNA extraction, firstly, potassium dichromate was removed from diluted oocyst samples through repeated centrifugation followed by resuspension in water. Secondly, the samples were subjected to repeated cycles of freezing, thawing and sonication using a specialized tissue homogenizer to crush the oocysts. Finally, genomic DNA was extracted according to the manufacturer’s protocol using the QIAamp stool DNA isolation kit (Qiagen, Germany). Eventually, the extracted DNA samples were kept at − 20 °C until further processing.

### ITS 1 PCR amplification and sequencing

**Fig. 2:**
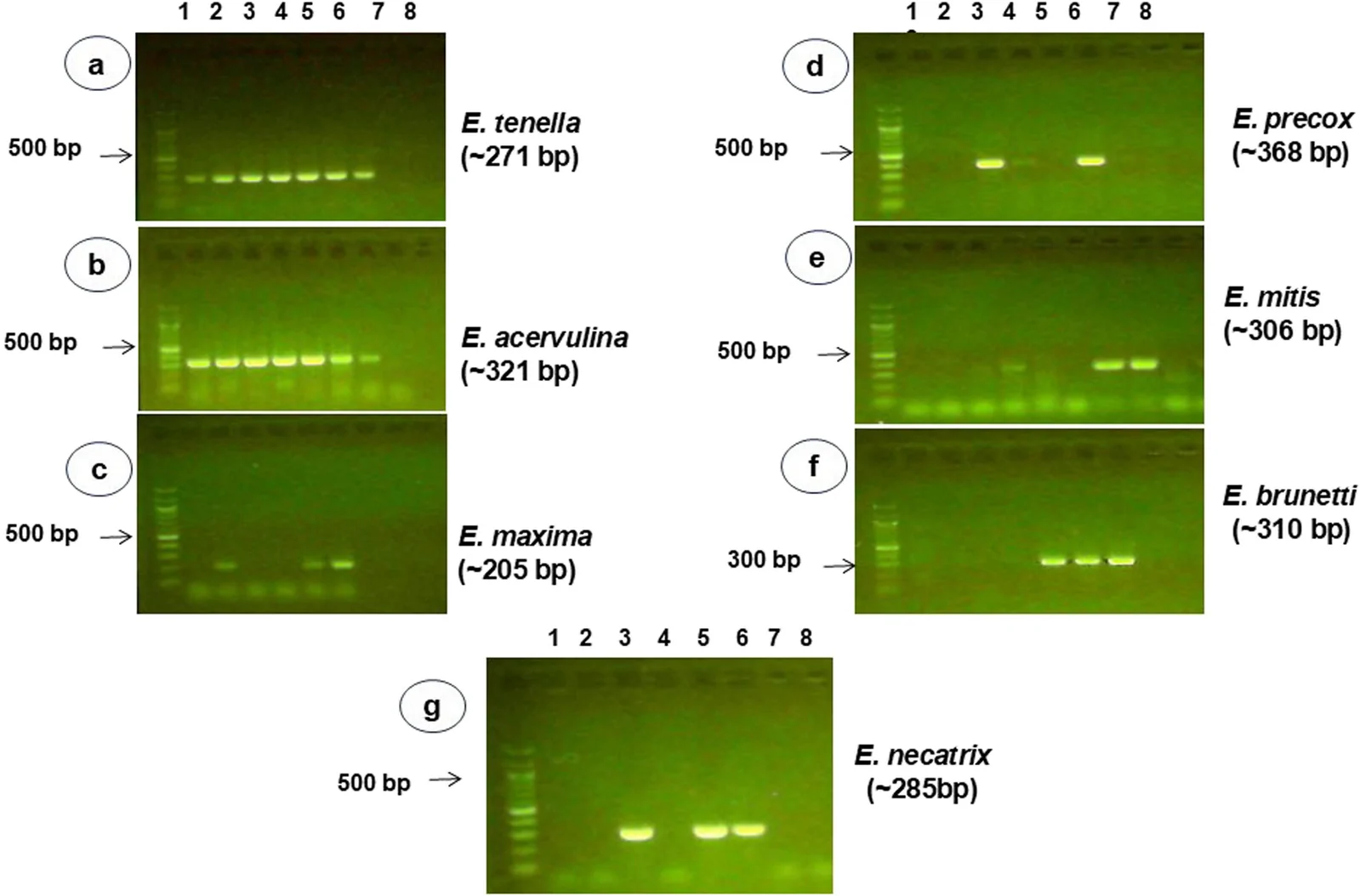
PCR products of Internal Transcribed Spacer 1 (ITS1) from *Eimeria* oocyst samples resolved on 1.5% agarose gel. The PCR product size was (**a**) E. tenella (~ 271 bp), (**b**) E. acervulina (~ 321 bp), (**c**) E. maxima (~ 205 bp), (**d**) E. necatrix (~ 285 bp), (**e**) E. precox (~ 368 bp), **f**) E. mitis (~ 306 bp), **g**) E. brunetti (~ 310 bp)

The extracted DNA was amplified using the ITS 1 (internal transcribed spacer 1) gene through the application of species-specific forward and reverse primer sets (Table 1) following the previously described protocol (Schnitzler et al. 1999). Following PCR amplification, all amplicons were assessed by electrophoresis through 1.5% agarose gel and subsequently visualized by SYBR safe (green) staining/Midori Green Advance DNA stain (Fig. 2). PCR-amplified fragments were subjected to Sanger sequencing using a commercially available automated sequencer. The representative sequences of each of *Eimeria* species were submitted to the GenBank for accession numbers. The respective accession numbers were displayed in Table 2. Prior to Sanger sequencing the PCR products were purified by using Qiagen QIAquick Kit.

**Table 1:** Primers used for the detection of *Eimeria* species in ITS 1 PCR assay

Species	Primer	Sequence (5’ − 3’)	Size(bp)	Annealing temp. (^o^C)	References
*E. maxima*	EmaF EMaR	GTGGGACTGTGG TGATGGGG ACCAGCATGCGCTCACAACCC	205	65	(Haug et al. 2007)
*E. praecox*	EPF EPR	CATCATCGG AATGGCTTTTTG A ATAAATAGCGCAAAATTAAGCA	368	54	(Jenkins et al. 2006)
*E. mitis*	EMiF EMiR	TAT TTCCTGTCGTCGTCTCGC GTATGCAAGAGAGAATCGGGA	306	54	
*E. acervulina*	EAF EAR	GGCTTGGATGATGTTTGCTG CGAACGCAATAACACACGCT	321	60	
*E. brunetti*	EBF EBR	GATCAGTTTGAGCAAACCTTCG TGGTCTTCCGTACGTCGG AT	310	45	
*E. necatrix*	ENF ENR	TACATCCCAATCTTTGAATCG GGCATACTAGCTTCGAGCAAC	285	55	
*E. tenella*	ETF ETR	AATTTAGTCCATCGCAACCCT CGAGCGCTCTGCATACGACA	271	60

**Table 2:** Univariate analysis of potential risk factors associated with the prevalence of *Eimeria* infection in chicken

Variables	Category (*n*)	Prevalence (%) (95% CI)	OR (95% CI)	*p*-value
Bird type	Broiler (202)	80.69 (75.2–86.1)	Reference 2.48 (1.27–4.85)	0.0076
	Sonali (148)	91.20 (86.7–95.8)		
Age	≤ 4 weeks (236)	82.62 (77.8–87.4)	Reference 1.97 (0.97–3.99)	0.0604
	> 4 weeks (114)	90.35 (85.0–95.8)		
Rearing system	Self (255)	83.92 (79.4–88.4)	Reference 1.46 (0.72–2.98)	0.294
	Contractual (95)	88.42 (81.9–94.8)		
Flock size	500–2000 (269)	87.36 (83.5–91.4)	1.97 (1.05–3.72) Reference	0.0358
	> 2000 (81)	77.77 (68.7–86.8)		
Previous history of coccidiosis	Yes (242)	91.67 (86.4–96.9)	2.38 (1.11–5.07) Reference	0.0251
	No (108)	82.23 (77.4–87.0)		
Bedding materials	Rice husk (152)	83.55 (77.7–89.5)	Reference 1.25 (0.69–2.25)	0.4642
	Saw dust (198)	86.36 (81.6–91.2)		
Type of floor	Packa (240)	83.33 (78.6–88.1)	Reference 1.63 (0.82–3.25)	0.1628
	Kacha (110)	89.09 (83.3–95.0)		
Source of drinking water	Tube-well water (159)	79.20 (68.0–81.1)	Reference 2.37 (1.28–4.36)	0.0055
	Tap water (191)	90.10 (85.8–94.3)		
Use of anticoccidials in the current flock	Yes (183)	89.60 (85.2–94.0)	2.13 (1.16–3.91) Reference	0.0152
	No (167)	80.20 (74.2–86.3)		
Use of disinfectants	Yes (159)	84.27 (78.6–90.0)	Reference 1.13 (0.63–2.04)	0.6778
	No (191)	85.86 (81.0–90.8)		
Consultation with	Veterinarian (104)	67.30 (58.3–76.3)	Reference 6.15 (3.27–11.56)	0.0001
	Non-vet. (246)	92.70 (89.4–95.9)		
Biosecurity status	Good (132)	81.80 (75.2–88.4)	Reference	-
	Moderate (136)	86.80 (80.7–94.8)	1.46 (0.75–2.83	0.2671
	Poor (82)	87.80 (81.1–92.5)	1.60 (0.72–3.55)	0.2470

### Analysis of sequence data

The forward and reverse sequences were trimmed using BioEdit software to generate consensus sequences. The identity percentage (%) of each of the study sequences was detected by the NCBI BLAST search, followed by comparison of the respective sequence with the reference sequence retrieved from the GenBank database (https://blast.ncbi.nlm.nih.gov/blast.cgi).

For phylogenetic analysis, two sequences from each of the studied species *E. acervulina*,* E. tenella*, *E. necatrix*,* E. maxima*,* E. mitis*,* E. brunetti and E. praecox*, five reference sequences from each of 7 *Eimeria* species (retrieved from the GenBank database by BLAST search identifying the most similar sites of the ITS1 gene), and one sequence of *Toxoplasma gondii* (EU025025.1) were aligned by ClustalW using MEGA11 (Tamura et al. 2021). A 50% bootstrap cut-off value was applied for the consensus tree (Alam et al. 2022), and all other parameters were set to default values in MEGA version 11.0. Finally, a Neighbor-Joining phylogenetic tree was constructed based on the Tamura-Nei model with a total of 50 sequences (Tamura et al. 2021).

### Epidemiological data analysis

All epidemiological data were analyzed in SPSS version 2025. Data on descriptive statistics such as bird type, age of birds, rearing system, flock size, previous history of coccidiosis, bedding materials, type of floor, source of drinking water, use of anticoccidials, use of disinfectants, consultation received, and biosecurity status were inserted in SPSS from the surveyed questionnaire and prevalence was calculated. Firstly, data were prepared for univariate analysis to estimate the strength and statistical significance of the associations between predictor variables and Eimeria infection. The variables that showed significant (p ˂0.05) in the univariate analysis were selected as potential candidates for multivariable analysis (multiple logistic regression with backward stepwise elimination). The p values for data inclusion and exclusion were set at 0.05 and ˃0.05, respectively. The final model was constructed with a significance level of p˂0.05 (Dey et al. 2020).

## Results

### Overall prevalence of *Eimeria* infection

**Fig. 3:**
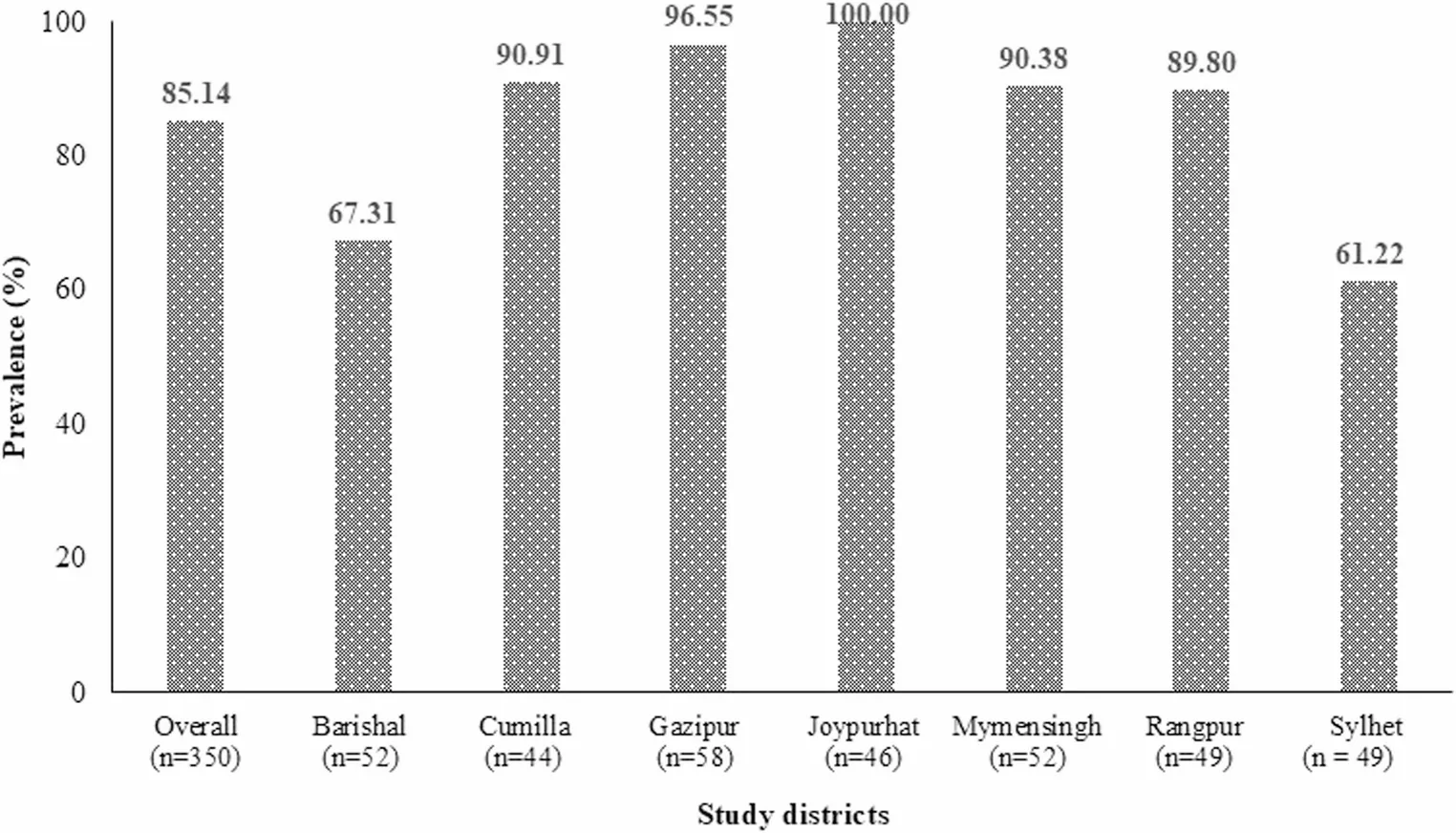
Overall and location wise prevalence of *Eimeria* infection

A total of 350 fecal samples were collected from commercial poultry farms across multiple districts of Bangladesh, of which 298 samples tested positive for *Eimeria* spp., yielding an overall prevalence of 85.14%. District-wise analysis revealed considerable variation in prevalence rates (Fig. 3). The highest prevalence of *Eimeria* infection was recorded in Joypurhat district (46/46; 100%), followed by Gazipur (56/58; 96.55%), Cumilla (40/44; 90.91%), Mymensingh (47/52; 90.38%) and Rangpur (44/49; 89.80%). In contrast, comparatively lower prevalence rates were recorded in Barishal (35/52; 67.31%) and Sylhet (30/49; 61.22%).

### Association between identified risk factors and coccidial infection

To explore the relationship of different epidemiological factors (birds’ age, bird type, flock size, previous history, source of water, litter used, use of disinfectants, use of anticoccidials, consultation service, etc.), a univariate analysis was performed (Table 2). The factors that were found significant in univariate analysis were subjected to multivariate analysis through stepwise logistic regression (Table 3). Univariate analysis revealed a significant association (*p* < 0.05) between the prevalence of *Eimeria* infection in broiler (80.69%) and sonali (92.22%) chicken with the odds ratio at 95% CI: 2.48 (1.27–4.85). A significant association (*p* < 0.05) was also detected between the age group of birds ≤ 4 weeks (82.62%) and >4weeks (90.35%) with an odds ratio at 95% CI: 1.97 (0.97–3.99). Farms with a previous history of coccidiosis, odds ratio: 1.25 (0.69–2.25) have significantly higher prevalence (91.67%) than those without a previous history of coccidiosis (82.23%). Besides, significantly higher prevalence was observed in the farms using tap water (90.1%) than that of using tube-well water (79.2%) (*p* < 0.05). Farms using anticoccidials after coccidiosis outbreak are more likely to have coccidiosis (89.6%) than those who did not have coccidiosis outbreak (80.2%) (*p* < 0.05). Veterinary consultation significantly decreases the prevalence (67.3%) of coccidiosis than consultation with the dealers or non-registered practitioners (92.7%), (*p* < 0.05) (Table 2). Among the statistically significant variables, the stepwise logistic regression model identified five variables of poultry farm practices, such as types of birds (*p* < 0.05), previous coccidiosis history (*p* < 0.05), water source (*p* < 0.05), use of anticoccidials (*p* < 0.05) and consultation with veterinarians (*p* < 0.05), as the associated risk factors of chicken coccidiosis.

**Table 3:** Multivariable analysis of possible risk factors related to chicken coccidiosis

Risk factors	OR	95% CI	*p*-value
Type of birds	2.487	1.00-6.15	0.049
Age of birds	2.111	0.80–5.52	0.128
Previous history of coccidiosis	17.252	5.49–54.14	0.000
Source of water	6.193	2.60-14.73	0.000
Use of anticoccidials	0.056	0.02–0.15	0.000
Consultation services	19.368	7.94–47.24	0.000

**Table 4:** Accession number of the respective sequences of different *Eimeria* species

Eimeria species	Given name	Accession number
*E. tenella*	BDMymEt01 BDMymEt16	PZ227117 PZ227118
*E. acervulina*	BDJoyEa10 BDMymEa24	PZ227119 PZ227120
*E. necatrix*	BDJOYEne40 BDJOYEne41	PZ227121 PZ227122
*E. maxima*	BDJOYEmax32 BDJOYEmax40	PZ227123 PZ227124
*E. mitis*	BDMymEmi16 BDGazEmi29	PZ227125 PZ227126
*E. brunetti*	BDJOYEbr32 BDJOYEbr40	PZ227127 PZ227128
*E. praecox*	BDMymEpr16 BDJOYEpr40	PZ227129 PZ227130

### Detection of *Eimeria* species by PCR

Amplification of the ITS 1 gene using species-specific primers followed by agarose gel electrophoresis, produced characteristic band sizes corresponding to different *Eimeria* species: *E. acervulina* (321 bp), *E. tenella* (271 bp), *E. necatrix* (285 bp), *E. maxima* (205 bp), *E. mitis* (306 bp), *E. brunetti* (210 bp), and *E. praecox* (368 bp). These results confirmed the presence of all seven *Eimeria* species in the field samples collected from Bangladesh (Fig. 2). BLAST search of specified sequences showed high nucleotide identity (98.08–100%) between study sequences and corresponding reference sequences retrieved from GenBank.

### Phylogenetic relationship of detected *Eimeria* species

**Fig. 4:**
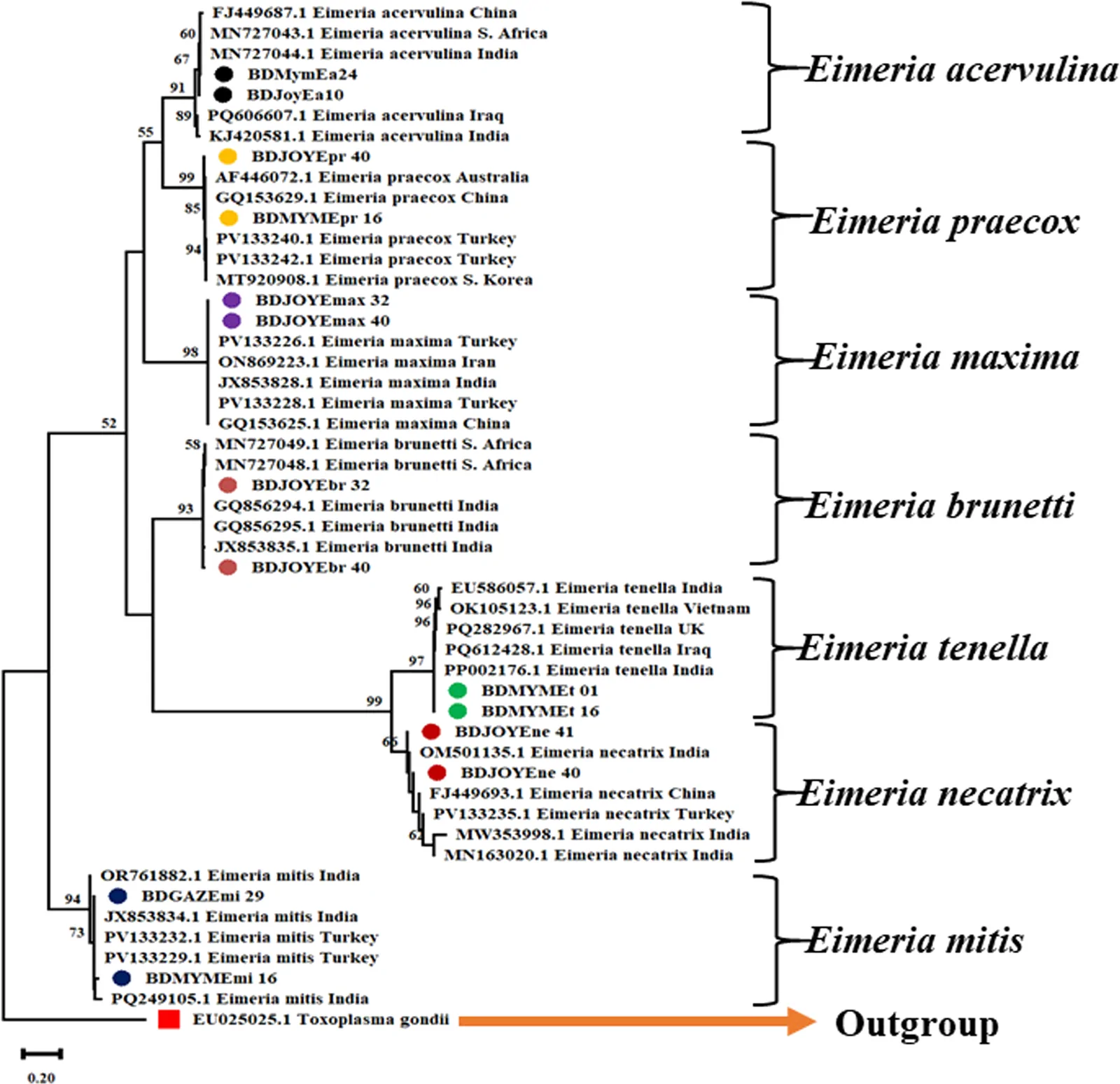
Neighbor joining phylogenetic tree of ITS1 gene sequences of different *Eimeria* spp (Tamura-3 model). The analysis involved 50 sequences (2 from each studied *Eimeria* spp, 5 reference sequences from each of 7 *Eimeria* spp. retrieved from GenBank and one sequence of *Toxoplasma gondii* as outgroup). Bootstrap values below 50% were not indicated. Percentage of bootstrap (1000 replicate) values is indicated in each node. The scale bar indicates sequence substitution per site. Evolutionary analyses were conducted in MEGA11. Studied sequences were presented as encircled (round colored), whereas out group was presented in rectangular shape (red colored)

Phylogenetic relationships were reconstructed using a Neighbor-Joining framework based on the aligned nucleotide sequences, with node robustness evaluated through 1,000 bootstrap replicates. The resulting topology is presented in Fig. 4. The analysis clearly positioned all study-derived sequences (designated with the prefix “BD” and further leveled with PZ as individual accession numbers (Table 4)) within established *Eimeria* lineages with high bootstrapping (52–99%). For instance, the studied *E. tenella* sequences (BDMymEt01-PZ227117 and BDMymEt16-PZ227118) formed clusters with their reference sequences with high bootstrap value (99%). Similarly, two isolates of *E. necatrix*, such as BDJOYEne40-PZ227121 and BDJOYEne41-PZ227122, were consistently assigned to the *E. necatrix* cluster. These sequences were grouped with reference isolates originating from India, China and Turkey, forming a compact subclade characterized by short internodal distances, indicative of high sequence similarity. In contrast, BDMymEmi16-PZ227125 and BDGazEmi29-PZ227126 were robustly placed within the *E. mitis* lineage (Fig. 4). This isolate clustered alongside reference sequences from India and Turkey, supported by comparatively higher bootstrap values (73–94%). No inconsistencies in clustering were observed across bootstrap replicates, and all BD isolates were unambiguously resolved at the species level. Collectively, the phylogeny delineates the presence of seven *Eimeria* species in the dataset.

### 3.5Occurrence of mixed infection

Out of 298 positive cases, 206 fecal samples were subjected to amplification of the ITS 1gene through PCR. It was revealed that mixed infections with more than one (2–7) *Eimeria* species were detected in 201 (97.57%) samples. All seven *Eimeria* species were recorded in only 3 (1.46%) cases. The highest prevalence of mixed infections (two *Eimeria* species) was recorded in 62 (30.10%) cases, with a peak of 52 (25.24%) cases involving a combination of *E. tenella* and *E. acervulina*. Co-infections with four *Eimeria* spp. were found in 57 (27.67%) samples, while infections with five species occurred in 48 (23.30%) cases. The second most common combination of *Eimeria* species (*E. tenella* + *E. acervulina + E. maxima + E. mitis + E. brunetti*) was recorded in 21 (10.19%) cases (Table 5).

**Table 5:** Rate of mixed infection in farm samples and combination of *Eimeria* spp. (*n* = 206)

No. of species	Name of Eimeria species	Frequency	Prevalence (%)	Cumulative (%)
One	EA	5	2.43	2.43
Two	ET + EA	52	25.24	30.10
	EA + EN	1	0.49	
	ET + EN	9	4.37	
Three	ET + EA+EP	13	6.31	7.28
	ET + EA+EMI	2	0.97	
Four	EA + EN+EB + EP	3	1.46	27.67
	ET + EA+EMI + EP	9	4.37	
	ET + EA+EN + EMI	3	1.46	
	ET + EA+EB + EP	3	1.46	
	ET + EA+EN+EMAX	15	7.28	
	ET + EA+EN + EP	12	5.83	
	ET + EA+EMAX + EMI	12	5.83	
Five	ET + EA+EN + EB+EP	6	2.91	23.30
	ET + EA+EMAX + EB+EP	6	2.91	
	ET + EA+EN+EMAX + EB	6	2.91	
	ET + EA+EN + EMI+EP	3	1.46	
	ET + EA+EMAX + EMI+EB	21	10.19	
	ET + EA+EMAX + EMI+EP	3	1.46	
	ET + EA+EN+EMAX + EP	3	1.46	
Six	ET + EA+EN+EMAX + EMI+EP	3	1.46	7.77
	ET + EA+EN + EMI+EB + EP	3	1.46	
	ET + EA+EN+EMAX + EB+EP	6	2.91	
	ET + EA+EN+EMAX + EMI+EB	4	1.94	
Seven	ET + EA+EN+EMAX + EMI+EB + EP	3	1.46	1.46
**Total**		**206**	**100**	**100**

Here, EA *Eimeria acervulina*, ET *Eimeria tenella*, EN *Eimeria necatrix*, EMAX *Eimeria maxima*, EMI *Eimeria mitis*, EB *Eimeria brunetti*, EP *Eimeria praecox*

## Discussion

Coccidiosis caused by *Eimeria* spp. remains a major constraint to poultry production due to its detrimental effects on intestinal integrity, growth performance, and overall economic return. A comprehensive understanding of prevalence, species distribution, mixed infection patterns, and associated risk factors is essential for elucidating disease epidemiology and improving control strategies.

The overall prevalence of *Eimeria* infection observed in this study was 85.14%, which is significantly higher than previously reported in Bangladesh, including 13.78% (Islam et al. 2025), 36% (Iqbal and Begum 2018) and 5.8% (Rahman et al. 2025). However, it remained slightly lower than reports from China, where prevalence rates above 97% have been documented (Geng et al. 2021; Liao et al. 2024). These discrepancies are likely due to differences in study design, geographic coverage, sampling strategies, bird age groups, and diagnostic methods. The inclusion of birds aged 15–60 days, considered highly susceptible to coccidiosis and the focus on intensive poultry production systems may explain the high prevalence found in this study. The findings align with reports from other Asian regions, including China, India, and Korea, suggesting similar epidemiological patterns under similar production conditions (Lee et al. 2010; Kumar et al. 2014; Huang et al. 2017). Significant spatial heterogeneity was evident, with prevalence ranging from 61.22% in Sylhet to 100% in Joypurhat. These differences probably reflect variations in environmental conditions, management practices, stocking density, and biosecurity measures. The higher prevalence in Joypurhat might be linked to the dominance of Sonali chickens, which are reported to be more susceptible to *Eimeria* infection (Mohammed and Obeta 2015; Prank et al. 2024). Such geographical differences highlight the importance of region-specific surveillance and tailored intervention strategies. Several farm-level risk factors were significantly associated with *Eimeria* infection, including bird type, water source, previous coccidiosis history, use of anticoccidials, and consultation practices. The higher infection rate observed in Sonali chickens (92.22%) supports earlier evidence of breed-related susceptibility (Islam et al. 2025). Farms using anticoccidials after disease outbreak are more likely to have oocysts in the fecal samples because once oocysts sheds in the litter can remain there for a long time for the persistence of favorable conditions. This finding is supported by Chapman and Johnson (1992). The link between tap water use and increased prevalence (90.1%) suggests inadequate water sanitation or contamination, emphasizing a key control point in farm management. The result is in accordance with the findings of Jubyda et al. (2021). Additionally, farms relying on non-registered service providers exhibited significantly higher infection rates (92.7%) compared to those with professional veterinary support (67.3%), underscoring the importance of trained expertise in disease prevention and control (Amare et al. 2012; Islam et al. 2020). This study is the first to report molecular detection of all seven recognized *Eimeria* species across seven districts of Bangladesh using an ITS 1-based PCR assay. When the study sequences were compared with reference sequences, BLAST search comparison depicted high nucleotide similarity (98.08–100%) among them. These high levels of sequence identity confirm the accurate molecular identification of the seven *Eimeria* species detected in this study. Furthermore, mixed infections with multiple *Eimeria* species pose challenges to coccidiosis control by increasing disease severity (Jenkins et al. 2008). The first report of mixed *Eimeria* infections in Bangladesh, involving 2–7 species, found in 97.57% of positive samples, aligns with other report (Liao et al. 2024) from China (93.25%). However, the result is significantly higher than the findings of Nasiri et al. (2024) (13.55%) and Gharekhani et al. (2014) (31.8%) from Iran. The increased prevalence of mixed *Eimeria* infections in the study farms may be predisposed by high stocking density, inadequate biosecurity measures and a favorable environment. Besides, the interactions among *Eimeria* species and their crowding effects could also be key factors in this regard (Williams 2001). Co-infections involving all seven *Eimeria* species were seen in 1.46% of positive samples, compared to 6.74% in China (Liao et al. 2024). Infection with only. *E. acervulina* was found in just 2.43% of samples, a finding comparable to that reported by Liao et al. (2024) (5.62%). The most frequent mixed infection involved two species (*E. acervulina* + *E. tenella*), detected in 25.24% of samples, followed by a five-species combination (*E. acervulina*, *E. tenella*, *E. maxima*, *E. mitis*, and *E. brunetti*) in 10.19% of cases. In contrast, Liao et al. (2024) reported the highest frequency of three and four-species mixed infections (20.22%) in China.

Phylogenetic analysis further supported species identification, where Bangladeshi isolates clustered with reference sequences and demonstrated high genetic conservation. Moderate bootstrap support observed in some clusters likely reflects limited variability within the ITS1 region rather than uncertainty in classification (Morris et al. 2007; Ogedengbe et al. 2011). In contrast, stronger support within the clade suggests differential resolution of the marker among species, consistent with previous findings (Clark et al. 2016; Ogedengbe et al. 2011). The co-occurrence of pathogenic (*E. tenella*, *E. necatrix*) and less virulent (*E. mitis*,* E. acervulina*) species highlights the potential for complex host–parasite interactions influencing disease outcomes. Additionally, the close phylogenetic relationship with isolates from neighboring countries suggests possible regional connectivity, potentially driven by similarities in production systems and cross-border movement. Building on these findings, future research integrating longitudinal designs, expanded geographic sampling, and advanced molecular tools such as multilocus genotyping or whole-genome sequencing would provide a more comprehensive view of population structure and transmission dynamics. Besides, combining quantitative infection data with host immune responses and farm management practices would further strengthen epidemiological modeling and inform targeted control strategies. Moreover, evaluating the field performance of anticoccidial programs and vaccination strategies would generate practical evidence to support improved and sustainable disease management.

## Conclusion

The overall high prevalence of *Eimeria* infection (85.14%) and its widespread geographic distribution indicate that coccidiosis remains endemic in poultry production systems in Bangladesh. This study highlights the evidence of all seven recognized *Eimeria* species infecting chickens. The extremely high rate of mixed *Eimeria* infection (97.57%) is of particular concern, as mixed infections are known to intensify disease severity, misguide diagnosis and negatively affect productive performance. Several factors, including rearing of Sonali chickens, use of tap water, previous history of coccidiosis outbreaks, use of anticoccidials, and reliance on non-registered veterinary service providers, were identified as important risk factors associated with chicken coccidiosis. Taking all into consideration, the high prevalence, widespread mixed infections, and multifactorial risk factors observed in this study emphasize the need for routine molecular surveillance and the implementation of effective anticoccidial shuttle programs. Importantly, the development of vaccines from locally circulating *Eimeria* strains and their application under field conditions could be a sustainable coccidiosis control strategy in Bangladesh.

## Supplementary Information

Below is the link to the electronic supplementary material.

Supplementary Material 1

## Data Availability

The data are, available upon request to corresponding author.
