# Supplementary material for: Prevalence, risk factors and mixed Eimeria infection detection in broiler and Sonali birds of some poultry concentrated zones of Bangladesh
Source: Parasitol Res. 2026 Jul 16;125(1):122. doi: 10.1007/s00436-026-08728-9 (PMC13654896; doi:10.1007/s00436-026-08728-9)
Supplement: Supplementary file 1 [file 436_2026_8728_MOESM1_ESM.docx]

**Project Name: Detection of anticoccidial drug resistance and development of vaccine candidate against chicken coccidiosis in Bangladesh**

Principal Investigator (PI):

Professor Dr. Mohammad Zahangir Alam

Dept. of Parasitology, Bangladesh Agricultural University, Mymensingh

**Questionnaire for data collection**

Note: Please put tick mark beside the appropriate answer. Where needed put the appropriate words/number

| **Serial No.** | **Question** | **Description** | | |
| --- | --- | --- | --- | --- |
| 1. | Farm name and number |  | | |
| 2. | Sample number as farm number |  | | |
| 3. | Farmers name |  | | |
| 4. | Farmers address | Upazila: | District: | |
| 5. | Mobile number of the farmers |  | | |
| 6. | Types of bird reared | Broiler | Sonali | |
| 7. | Rearing system | Self | Contact farming | |
| 8. | Age of Bird (during survey) (Days) |  | | |
| 9. | Flock size | 500-2000 | 2000-above | |
| 10. | Did you face coccidiosis in your farm? | Yes | No | |
| 11. | Bedding materials used | Rice husk | Saw dust | Others |
| 12. | Do you use old litter as bedding material? | Yes | No | |
| 13. | Do you use old litter? | Yes | No | |
| 14. | How often do you stirrer the litter daily? | 2 times | One time | Not at all |
| 15. | Time interval between two batches |  |  |  |
| 16. | What type of feed do you supply to the birds? | Self-made | Ready made |  |
| 17. | What is the source of drinking water | Tubewell | Tap water | others |
| 18. | Do you use anticoccidials in the current batch? | Yes | No |  |
| 19. | Do you use disinfectants? | Yes | No |  |
| 20. | How do you dispose farm litter after each batch? | Near the farm | Far away from the farm | Others |
| 21. | Do you rear different aged birds in the same shed? | Yes | No |  |
| 22. | To whom do you consult when face trouble? | Veterinarian | Non- vet service providers | Self |
| 23. | Biosecurity status of the farm | Good | Moderate | Poor |

Name & Signature of data collector

Date:
